# SNX10 regulates the proliferation, apoptosis and cell cycle of acute B lymphoblastic leukemia cells via the PI3K/Akt signaling pathway

**DOI:** 10.3892/or.2025.8911

**Published:** 2025-05-08

**Authors:** Chenyu Wang, Xiulan Yang, Xue Shen, Shirong Yan, Jing Li, Yan Wang, Tian Tao, Tongqian Wu, Qian Kang, Fang Yu

**Affiliations:** 1 Center for Clinical Laboratories, Affiliated Hospital of Guizhou Medical University, Guiyang, Guizhou 550004, P.R. China; 2 Department of Clinical Microbiology and Immunology, School of Clinical Laboratory Science, Guizhou Medical University, Guiyang, Guizhou 550004, P.R. China; 3 Clinical Research Center, Affiliated Hospital of Guizhou Medical University, Guiyang Guizhou 550004, P.R. China; 4 Department of Hematology, Affiliated Hospital of Guizhou Medical University, Guiyang, Guizhou 550004, P.R. China

**Keywords:** B-cell acute lymphoblastic leukemia, sorting nexin 10, PI3K/Akt signaling pathway, proliferation, apoptosis, cell cycle, bioinformatics analysis

## Abstract

B-cell acute lymphoblastic leukemia (B-ALL) is a type of acute lymphoblastic leukemia that originates from B cells. It typically occurs in children and adolescents, but it can also appear in adults. Sorting nexin 10 (SNX10) has recently been identified as a significant regulatory factor in various tumors, although its specific roles remain contested. However, its function in B-ALL has not been previously explored. The present study investigated the role of SNX10 in B-ALL pathogenesis. Bioinformatics analysis identified SNX10 as a Core Hub gene in the B-ALL signaling network, with significantly reduced expression in patients with B-ALL. These findings were corroborated through analysis of clinical bone marrow samples and B-ALL cell lines. Functional *in vitro* studies revealed that SNX10 knockdown markedly inhibited B-ALL cell proliferation, increased apoptosis, and arrested cells in the G0/G1 phase. By contrast, SNX10 overexpression enhanced cell proliferation, suppressed apoptosis and promoted G2/M phase progression. Proteomic analysis further implicated the PI3K/Akt signaling pathway in mediating the effects of SNX10. Specifically, SNX10 overexpression increased the phosphorylation levels of PI3K and Akt, while SNX10 knockdown had the opposite effect. *In vivo* experiments demonstrated that elevated SNX10 expression accelerated leukemia progression in a mouse model. Collectively, these findings highlighted the pivotal role of SNX10 in promoting B-ALL cell proliferation via the PI3K pathway, highlighting its potential as a therapeutic target for B-ALL and providing a foundation for future investigations.

## Introduction

B-ALL is a hematologic malignancy caused by the proliferation of malignant B lymphoblast clones (1), predominantly affecting children and adolescents (2), but also observed in adults (3). Treatment strategies for B-ALL include chemotherapy (4), targeted therapy (5) and immunotherapy (6), which together improve remission rates and overall outcomes. Although >90% of patients achieve initial complete response through chemotherapy, up to 50% of patients with B-ALL experience a relapse, developing chemotherapy-resistant disease (7). Immunotherapy has shown promise in reducing relapse by effectively targeting minimum residual disease, but its efficacy remains limited due to the intrinsically low immunogenicity of B-ALL cells (7). Therefore, identifying novel therapeutic targets to enhance treatment response and improve long-term survival remains a critical priority in B-ALL research.

In 2023, Krali *et al* (8) developed a machine learning algorithm to patients with subtype pediatric B-ALL based on a molecular profile, generating the GSE227832 dataset. This dataset includes gene expression profiles from 315 newly diagnosed cases, 8 relapse cases, 10 healthy donors, and 2 cases in remission. By analyzing the GSE227832 dataset in combination with the TARGET-ALL-P3 dataset [from The Cancer Genome Atlas (TCGA)] including clinical data from 79 patients, three core hub genes closely associated with the progression of B-ALL were identified: Glucose-dependent insulinotropic polypeptide receptor (GIPR), hepatocyte growth factor (HGF) and sorting nexin 10 (SNX10). These genes, all linked to disease progression, present promising targets for further investigation into the molecular mechanisms driving B-ALL. In the present study, a focus was placed on SNX10 due to its unique and previously unexplored role in hematologic malignancies, particularly in B-ALL.

SNX10 is a member of the phosphoinositide-binding protein family and contains a phox homology (PX) domain, facilitating intracellular protein transport through interaction with phosphoinositide-enriched membranes (9). Known to be highly expressed in bones and intestines, SNX10 regulates various physiological processes, including osteoclast bone resorption (10), gastric acid secretion (11) and glucose metabolism (12). Increasing evidence suggests that SNX10 also plays complex roles in cancer. For example, SNX10 has demonstrated tumor-suppressive effects in colorectal cancer (13), driving inflammation-related colorectal cancer through a chaperone-mediated autophagy mechanism. By contrast, elevated SNX10 expression in cervical cancer has been associated with increased invasion and metastasis (14), and this is associated with a poor prognosis in patients with glioblastoma (15) suggesting that the role of SNX10 in cancer is context-dependent. These findings underscore the dual role that SNX10 may play in cancer biology, likely influenced by tissue type and cellular context. Despite mounting evidence linking SNX10 to various aspects of cancer, its role in B-ALL remains undetermined, to the best of our knowledge. Given the regulatory functions of SNX10 in intracellular transport and signaling, it is plausible that it could influence B-ALL pathophysiology by interacting with key signaling pathways.

The PI3K/Akt signaling pathway is a critical cell signaling pathway involved in various biological processes such as cell survival (16), proliferation (17), differentiation (18), migration (19) and metabolism (20), and is well-established to be implicated in various hematologic malignancies, including leukemia (21). Typically activated by growth factors (22) or signaling molecules binding to cell membrane receptors, this pathway triggers a cascade leading to PI3K activation and subsequently Akt phosphorylation. p-Akt then regulates cell cycle progression through the mTOR pathway (23) and inhibits apoptosis by modulating Bcl-2 family proteins (24). Additionally, the PI3K/Akt/PIKfyve/PtdIns(3,5)P2 signaling pathway is crucial for viral entry into cells (25), with PIKfyve, which phosphorylates PI3P to PI(3,5)P2, colocalizing with Snx10 in early endosomes of osteoclasts to regulate endosome/lysosome homeostasis (26). However, the potential relationship between SNX10 and the PI3K/Akt signaling pathway in B-ALL has not been previously examined.

In the present study, the role of SNX10 in B-ALL was examined. Using systematic *in vitro* and *in vivo* experiments, it was revealed that SNX10 impacted B-ALL proliferation, apoptosis and migration by modulating the PI3K/Akt signaling pathway. These findings provide novel insights into the involvement of SNX10 in B-ALL progression, addressing previously unexplored questions regarding its function in leukemia and highlighting potential therapeutic avenues for the management of B-ALL.

## Materials and methods

### Screening of common differentially expressed genes (DEGs) in B-ALL

The GSE227832 dataset was downloaded from the Gene Expression Omnibus (GEO) database (https://www.ncbi.nlm.nih.gov/geo/) for differential analysis. Subtypes of B-ALL with at least 10 samples were selected. The subtypes included were ALLIUM B-other, BCR:ABL1, BCR:ABL1-like, DUX4-r, ETV6:RUNX1, ETV6:RUNX1-like, HeH, KMT2A-r, PAX5alt, TCF3:PBX1, ZNF384-r and iAMP21. The limma package (https://bioconductor.org/packages/release/bioc/html/limma.html) was used to calibrate and normalize the raw data in R (https://www.r-project.org/), and then DEGs in each subtype were selected using the criteria |log_2_FC|<1.5 and P.adjust <0.05, separately. Finally, a basic function in R, the intersect function was used to find the common DEGs in the 12 different subtypes of B-ALL.

### Screening for survival-associated DEGs

The gene expression profiles and clinical data for 79 patients were retrieved from TCGA (https://portal.gdc.cancer.gov/projects/TARGET-ALL-P3). Samples with gene expression levels above the mean were categorized into the high expression group (H), whereas those at or below the average were categorized into the low expression group (L). To identify survival-associated DEGs (Survdiffs), a log-rank test from the survival Package (https://cran.r-project.org/web/packages/survival/index.html) was performed to compare survival rates between the two groups in R. Subsequently, the survfit function from the survival package was used to fit a Kaplan-Meier survival curve. Finally, the survival time curve analysis was visualized using the ggsurvplot function from the survminer package (https://cran.r-project.org/web/packages/survminer/index.html).

### Identification of core hub genes in B-ALL

The Least Absolute Shrinkage and Selection Operator (LASSO) is a linear regression regularization method used for feature selection and model simplification (27). LASSO shrinks certain regression coefficients to zero, retaining only variables with non-zero coefficients within the model. In the present study, common DEGs and Survdiffs were imported into R. The intersect function was used to find the intersection of the common DEG sets and Survdiffs sets, and the ggvenn Package (https://cran.r-project.org/web/packages/ggvenn/index.html) was used for visualization. LASSO analysis with the glmnet package (https://cran.r-project.org/web/packages/glmnet/index.html) was used to refine these intersecting genes and identify the Core Hub genes. Finally, a Cox proportional hazards regression model was calculated using the coxph function in the survival package, and the survminer package was used to summarize and visualize the results of the survival analysis.

### Research population and sample

The subjects recruited for the present included 24 patients with newly diagnosed B-ALL at the Affiliated Hospital of Guizhou Medical University (Guiyang, China) between May 2023 and September 2024, and 24 age-matched healthy controls (HC). The age range of the patients was 1–79 years, a median age of 15 years, and including 10 males and 14 females. Written informed consent was obtained from all participants or their legal guardians for those under the legal age of consent. The study was approved by the Ethics Committee of the Affiliated Hospital of Guizhou Medical University (approval no. 2022328). The procedures performed in the present study strictly adhered to the ethical principles outlined in the Declaration of Helsinki (28). The patient's clinicopathological information is shown in Table SI. Bone marrow mononuclear cells (BMMCs) were isolated from bone marrow specimens of patients with B-ALL and normal healthy donors using Ficoll density centrifugation kit, according to the manufacturer's protocol (Beijing Solarbio Science & Technology Co., Ltd.). Briefly, an equal volume of PBS was added to the bone marrow sample, mixed gently, and then carefully layered onto a tube that contained 3 ml Ficoll separation reagent. The mixture was centrifuged at 400 × g for 35 min at room temperature, and the middle white cell layer, which was the mononuclear cell layer, was gently aspirated.

### Cell lines

The human B-ALL Nalm-6 (cat. no. CC-Y1706) and Rs4;11 (cat. no. CC-Y1741) cells (both from Shanghai EK-Bioscience Biotechnology Co., Ltd.) were cultured in RPMI-1640 medium containing 10% FBS (both from Gibco; Thermo Fisher Scientific, Inc) and 1% penicillin-streptomycin at 37°C in a humidified incubator supplied with 5% CO_2_ air. 293T cells (Pricella Biotechnology Co., Ltd.) were cultured in DMEM medium (Gibco; Thermo Fisher Scientific, Inc.) supplemented with 10% FBS and 1% penicillin-streptomycin at 37°C in a humidified incubator supplied with 5% CO_2_ air. The identity of these cell lines was genetically confirmed by short tandem repeat analysis and was routinely tested for mycoplasma contamination.

### Reverse transcriptase-quantitative PCR (RT-qPCR)

According to the manufacturer's protocol, total RNA was extracted from BMMCs using the R6834 Total RNA extraction kit (Omega Bio-Tek, Inc.). A total of 1 µg RNA was reverse transcribed into cDNA using a PrimeScript RT-PCR kit, according to the manufacturer's protocol (Shanghai Yeasen Biotechnology Co., Ltd.). cDNA, SYBR Green PCR Master Mix kit (Shanghai Yeasen Biotechnology Co., Ltd.), and gene-specific primers for SNX10 were used for qPCR on a Gentier 96E fully automated medical PCR analysis system (Xi'an Tianlong Technology Co., Ltd.). All reactions were conducted in a 20 µl reaction volume in triplicate. Primers were synthesized by Sangon Biotech Co., Ltd. The sequences of the primers were: SNX10 forward, 5′-CACTTTTGCTTTCAGATAGCAGC-3′ and reverse, 5′-ACACACGCCTCAATGTCTTCT-3′, and β-actin forward, 5′-CCTGGCACCCAGCACAAT-3′ and reverse, 5′-GGGCCGGACTCGTCATAC-3′. The thermocycling conditions were: Initial denaturation for 2 min at 95°C, followed by 40 cycles of 95°C for 10 sec and 60°C for 30 sec. After the reaction was completed, the relative mRNA expression levels were normalized to β-actin and calculated using the 2^−ΔΔCq^ method (29).

### Western blotting

Following the manufacturer's guidelines, proteins were extracted using RIPA lysis buffer (Beijing Solarbio Science & Technology Co., Ltd.) with 1 mmol/l PMSF and 1% Phosphatase Inhibitor Cocktail (×100) (both from Shanghai Epizyme Biomedical Technology Co., Ltd.). Protein concentrations were estimated using an Omni-Easy^™^ Instant BCA Protein Assay Kit (Shanghai Epizyme Biomedical Technology Co., Ltd.). Equal quantities of protein extracts (30 µg) were loaded on 10% SDS-gels, resolved using SDS-PAGE, and transferred to PVDF membranes (MilliporeSigma). The membranes were blocked with 5% BSA (Beyotime Institute of Biotechnology) for 2 h at room temperature, followed by overnight incubation with primary antibodies at 4°C. The following day, membranes were washed and incubated with the HRP-conjugated secondary antibody (1:10,000; cat. no. SA00001-1 or cat. no. SA00001-2; Proteintech Group, Inc.) at 37°C for 1 h. Membranes were washed again with TBS-0.1% Tween three times, and signals were visualized using an enhanced chemiluminescent reagent (Dalian Meilun Biotechnology Co., Ltd.) and observed using an ECL chemiluminescence system (Bio-Rad Laboratories, Inc.). Densitometric analysis was performed using ImageJ (version no.1.53; National Institutes of Health) and normalized to the loading control (β-actin). The following antibodies were used: Anti-SNX10 (1:1,000; cat. no. MG928896S), anti-kinase p85-α (1:1,000; cat. no. A31285), anti-phospho-PI3-kinase p85-α/γ (1:1,000; cat. no. T40116) all from Abmart Shanghai Co., Ltd.; anti-Akt(pan) 1/2/3 (1:1,000; cat. no. A75065), anti-P-Akt (1:1,000; cat. no. A52820) from Nature biosciences Co., Ltd.; and anti-human β-actin (1:10,000; cat. no. AC026) from ABclonal Biotech Co., Ltd.

### Plasmid and lentivirus infection

SNX10 overexpression plasmid (OE), empty vector plasmid (EV), SNX10 short hairpin plasmid (shSNX10), and the negative control (shNC), all plasmids labeled with FITC fluorescence, were purchased from Guangzhou IGE Biotechnology Co., Ltd. The SNX10 OE plasmid and shSNX10 sequences are shown in Table SII. For transfection, 500 µl Opti-MEM medium was added into each of two 1.5 ml EP tubes. Then, 10 µl Lipo8000™ transfection reagent (Beckman Coulter, Inc.) and a plasmid mixture (the ratio of target plasmid to packaging plasmids psPAX2 and pMD2.G was 1.0:0.5:1.0 µg) were added. The plasmids were mixed separately and left to stand for 5 min. Following this, the Lipo8000^™^ transfection reagent was added to the plasmid mixture, gently mixed, and allowed to sit for 20 min prior to transfection into 293T cells that were cultured in a 60-mm dish at 80% confluence (30). A total of 6–8 h after transfection, the medium was replaced, and the viral supernatant was collected after 24, 48 and 72 h, and filtered through a 0.45-µm filter membrane. To concentrate every 20 ml of viral supernatant, 5.5 ml of 44% PEG8000 (Beijing Solarbio Technology Co., Ltd.) and 2 ml (4 mol/l) NaCl were added, mixed, and incubated overnight at 4°C. After 24 h, the mixture was centrifuged at 3,500 × g for 15 min, and the pellet was resuspended in 150 µl PBS. A total of 50 µl of the concentrated virus solution was then added to Nalm-6 or RS4:11 cells (5×10^6^ cells) in the presence of 5 µl (0.8 µg/ml) polybrene (Beijing Solarbio Science & Technology Co., Ltd.). The culture medium was replaced 6–8 h after infection, and the cells were cultured for 7 days. Stable cell lines were selected using puromycin (Beijing Solarbio Science & Technology Co., Ltd.) at concentrations of 1.5 µg/ml for Nalm-6 cells and 2.0 µg/ml for RS4:11 cells (31). The protein and mRNA expression levels were assessed using western blotting and RT-qPCR, respectively.

### Pathway inhibition assays

Idelalisib (IDEL, TargetMoi, Cas: 870281-82-6) is a small molecule inhibitor that targets the PI3K catalytic subunit p110δ. A 10 mM stock solution was prepared by dissolving it in DMSO and stored at −20°C. Subsequently, 2 ml of 5×10^5^ transfected cells/ml was seeded into a 6-well plate, and IDEL was added to achieve a final concentration of 10 µM (32). In the vehicle control group, an equivalent volume of 0.1% DMSO was used as the solvent control group. After gently mixing, the cells were incubated for 24 h for subsequent detection.

### Cell proliferation assay

According to the manufacturer's protocol, the transfected cells were harvested and diluted to a concentration of 3×10^5^ cells/ml. A total of 100 µl (3×10^4^ cells) was added to each well of a 96-well plate, with three replicates per condition. After 0, 24, 48 and 72 h of incubation, 10 µl Cell Counting Kit-8 solution (Dalian Meilun Biotechnology Co., Ltd.) was added to each well, followed by an additional 2 h of incubation. At each time point, the absorbance at 450 nm was measured using a microplate reader (Thermo Fisher Scientific, Inc.).

### Cell apoptosis assay

According to the manufacturer's guidelines, the transfected cells were harvested and diluted to a concentration of 1×10^6^ cells/ml with 1X Binding Buffer (Pricella Biotechnology Co., Ltd.). A total of 100 µl of the cell suspension (1×10^5^ cells) was transferred to a new tube, and 5 µl Annexin V-APC and 5 µl Cyanine7/7-AAD (Pricella Biotechnology Co., Ltd.) were added. The solution was gently mixed and incubated in the dark at room temperature for 15 min. Following incubation, 400 µl 1× Binding Buffer was added. Finally, these cells were analyzed using a flow cytometer (NAVIOS; Beckman Coulter Inc.). The experimental data were processed using FlowJo software (v10.8.1, FlowJo LLC).

### Cell cycle analysis

According to the manufacturer's guidelines, the transfected cells were harvested and diluted to a concentration of 1×10^6^ cells/ml. A total of 1 ml of the suspension was taken, the supernatant was removed by centrifugation at 150 × g for 5 min, and then resuspended in 500 µl pre-cooled 70% ethanol. The cells were fixed at 4°C overnight and then washed with PBS to remove the supernatant. The cells were resuspended in a mixture of 500 µl RNase A and PI (in a 1:9 ratio) and incubated in the dark at 37°C for 30 min. Analysis was then performed using a flow cytometer (NAVIOS; Beckman Coulter Inc.). The experimental data were processed using ModFit software (v3.1; Verity Software House).

### Quantitative proteomics

To extract total protein, 5×10^6^ cells were lysed with 300 µl lysis buffer (8 M urea, 1 mM PMSF, 2 mM EDTA), then sonicated on ice for 5 min. The sample was then centrifuged at 4°C for 10 min at 15,000 × g, and the supernatant was collected. The protein concentration was determined using a BCA kit (Beyotime Institute of Biotechnology). A total of 100 µg of the protein solution was received and the volume was adjusted to 200 µl with 8 M urea. The solution was reduced with 5 mM DTT at 37°C for 45 min, followed by alkylation with 11 mM iodoacetamide in a dark room at room temperature for 15 min. Subsequently, 800 µl 25 mM ammonium bicarbonate solution and 3 µl trypsin (Promega Corporation) were added, and digestion was performed overnight at 37°C. The pH of the digested peptides was adjusted to 2–3 using 20% trifluoroacetic acid, and desalination was performed using C18 resin (MilliporeSigma). Finally, the peptide concentration was determined using the Pierce^™^ Quantitative Peptide Detection Reagent and Standard Kit (Thermo Fisher Scientific, Inc.). Chromatographic separation was performed using the nanoscale Vanquish Neo system (Thermo Fisher Scientific, Inc.), and the samples were separated by nanoscale high-performance liquid chromatography and analyzed using an Orbitrap Astral high-resolution mass spectrometer (Thermo Fisher Scientific, Inc.) for data-independent acquisition mass spectrometry analysis. Finally, Kyoto Encyclopedia of Genes and Genomes (KEGG) pathway enrichment analysis was performed using the enrichKEGG function and the KEGG enrichment results were visualized using a barplot in R. The mass spectrometry proteomics data have been deposited to the ProteomeXchange Consortium (https://proteomecentral.proteomexchange.org) via the iProX partner repository (33,34) with the dataset identifier PXD061830.

### Cell-derived xenograft (CDX) mouse model

A total of 20 female NOD-SCID mice were purchased from Beijing Vital River Laboratory Animal Technology Co., Ltd., aged 8–12 weeks and weighing ~22 g, and were randomly divided into the SNX10 OE group (n=10) and the EV group (n=10), and were housed in the SPF laboratory of the Affiliated Hospital of Guizhou Medical University. When cells reached the logarithmic phase of growth, they were collected and counted. A total of 5×10^6^ transfected cells in 200 µl PBS were injected through the tail vein and the mice were monitored for 60 days. The body weight and the dates of onset and death were recorded. On day 21 post-injection, five mice were randomly selected from each group, and bone marrow, peripheral blood, liver, spleen and lymph nodes were collected for analysis. At the end of the experimental period, mice were euthanized by cervical dislocation under 2.0–2.5% isoflurane inhalation anesthesia. Death was confirmed by monitoring of vital signs to confirm death. The animal experiments were approved by the Guizhou Medical University Animal Care Committee (approval no. 2402141).

### Flow cytometry analysis

On the 21st day post-injection, five mice from each group were randomly selected for euthanasia, and bone marrow, peripheral blood, and lymph node tissues were collected to prepare a single-cell suspension. Following the manufacturer's guidelines, red blood cells were lysed using a red blood cell lysis buffer (Gibco; Thermo Fisher Scientific, Inc), and the remaining cells were resuspended in 100 µl sheath fluid. The cells were then incubated in the dark at 4°C for 15 min with the addition of 5 µl anti-human CD19 antibody (cat. no. 4058218; BD Biosciences). Following the incubation, 400 µl sheath fluid was added. The human B-ALL cells (human CD19-APC/GFP-FITC) were identified using a flow cytometer (BD CANTO PLUS; BD Biosciences).

### Hematoxylin and eosin (H&E) staining

On the 21st day of the experiment, the liver and spleen of the euthanized mice were collected. The weight and volume of the liver and spleen were recorded. According to the manufacturer's guidelines, the tissue samples were fixed with 4% formaldehyde (Beijing Solarbio Science & Technology Co., Ltd.) for 24 h at room temperature, followed by dehydration, clearing, and infiltration with paraffin to create paraffin-embedded blocks. These blocks were then cut into 4-m thick sections and placed on slides. The slides were immersed in xylene for deparaffinization twice, each for 5 min. Dehydration was performed using 100, 95, 90 and 80% ethanol solutions (5 min each), followed by a rinse with water. The sections were stained in hematoxylin solution (Wuhan Siwega Biotechnology Co., Ltd.) for 15 min at room temperature, rinsed, and then differentiated in 1% hydrochloric acid alcohol for 1 min, after which they were rinsed with water. The sections were subsequently immersed in eosin solution (Wuhan Siwega Biotechnology Co., Ltd.) for 5 min, rinsed, and dehydrated with 80, 90, 95, and 100% ethanol solutions (5 min each). Finally, the sections were cleared using xylene, immersed in mounting medium, and a coverslip was placed on top. The tissues were observed under a microscope and underwent electronic scanning (Pannoramic MIDI, 3DHISTECH Ltd.).

### Statistical analysis

R and GraphPad Prism version 9.0 (GraphPad Software Inc.; Dotmatics) were used for statistical analyses. An unpaired Student's t-test was used to compare differences between two groups. For comparisons between multiple groups, a one-way ANOVA or two-way ANOVA were used and followed by Tukey's multiple comparisons test. Survival data were plotted using Kaplan-Meier survival curves, and a log-rank test was used to compare differences in survival. P<0.05 was considered to indicate a statistically significant difference.

## Results

### GIPR, HGF and SNX10 are core hub genes in B-ALL

**Figure 1.**
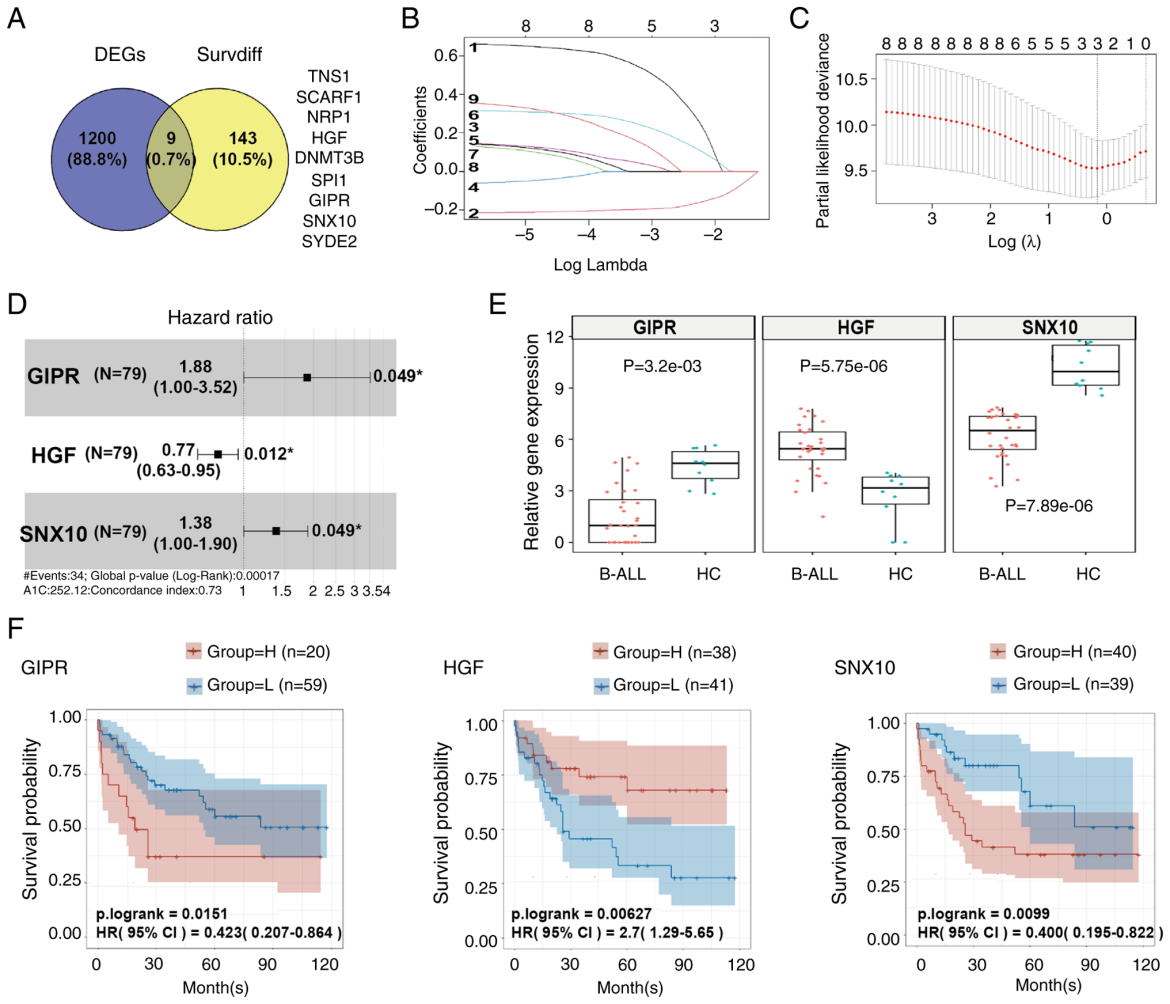
GIPR, HGF and SNX10 are confirmed as core hub genes in B-ALL. (A) Venn diagram illustrating the nine overlapping genes in common DEGs and Survdiffs. (B) LASSO coefficient path diagram of nine risk factors. (C) Cross-validation curves of GIPR, HGF and SNX10. (D) Cox proportional hazards model of GIPR, SNX10 and HGF. (E) Box plots showing the gene expression levels of GIPR, HGF, and SNX10 in patients with B-ALL (n=32) and the HC group (n=10). (F) Kaplan-Meier survival analysis curves for GIPR, HGF and SNX10. B-ALL, B-cell acute lymphoblastic leukemia; HC, healthy control; GIPR, gastric inhibitory polypeptide receptor; HGF, hepatocyte growth factor; SNX10, sorting nexin 10; DEG, differentially expressed gene; Survdiff, survival-associated differentially expressed gene; LASSO, Least Absolute Shrinkage and Selection Operator; HC, healthy control.

A dataset containing samples of 12 B-ALL subtypes was downloaded from GEO and analyzed. A total of 1,209 common DEGs were identified (Table SIII, Fig. 1A). Concurrently, another dataset from TCGA was obtained and analysis of this dataset showed 152 survival-associated DEGs (Table SIV, Fig. 1A). Using a Venn diagram, the intersection of these DEGs showed nine common genes: TNS1, SCARF1, NRP1, HGF, DNMT3B, SPI1, GIPR, SNX10 and SYDE2 (Table SV, Fig. 1A). Through LASSO regression analysis, GIPR (LASSO coefficient=0.2062), HGF (LASSO coefficient=−0.1533) and SNX10 (LASSO coefficient=0.0945) were confirmed as hub genes within B-ALL protein network (Fig. 1B and C), indicating their potential regulatory and functional significance in B-ALL biology. Furthermore, results from the Cox proportional hazards model indicated that high expression levels of GIPR (HR=1.88, P=0.049), SNX10 (HR=1.38, P=0.049), and low expression of HGF (HR=0.77, P=0.012), were independent adverse prognostic factors for overall survival (OS) in patients with B-ALL (Fig. 1D). Boxplot analyses indicated that compared with the HC group, the expression of GIPR and SNX10 was downregulated in patients with B-ALL, whereas HGF expression was upregulated (Fig. 1E). Kaplan-Meier survival curves further supported these findings, showing that patients with low expression of GIPR and SNX10 had a higher OS rate, whereas those with high expression levels exhibited a lower OS rate. Conversely, high expression of HGF is linked to a higher OS rate, and low expression was correlated with a poorer survival outcome (Fig. 1F).

### SNX10 expression is decreased in B-ALL specimens and cell lines

**Figure 2.**
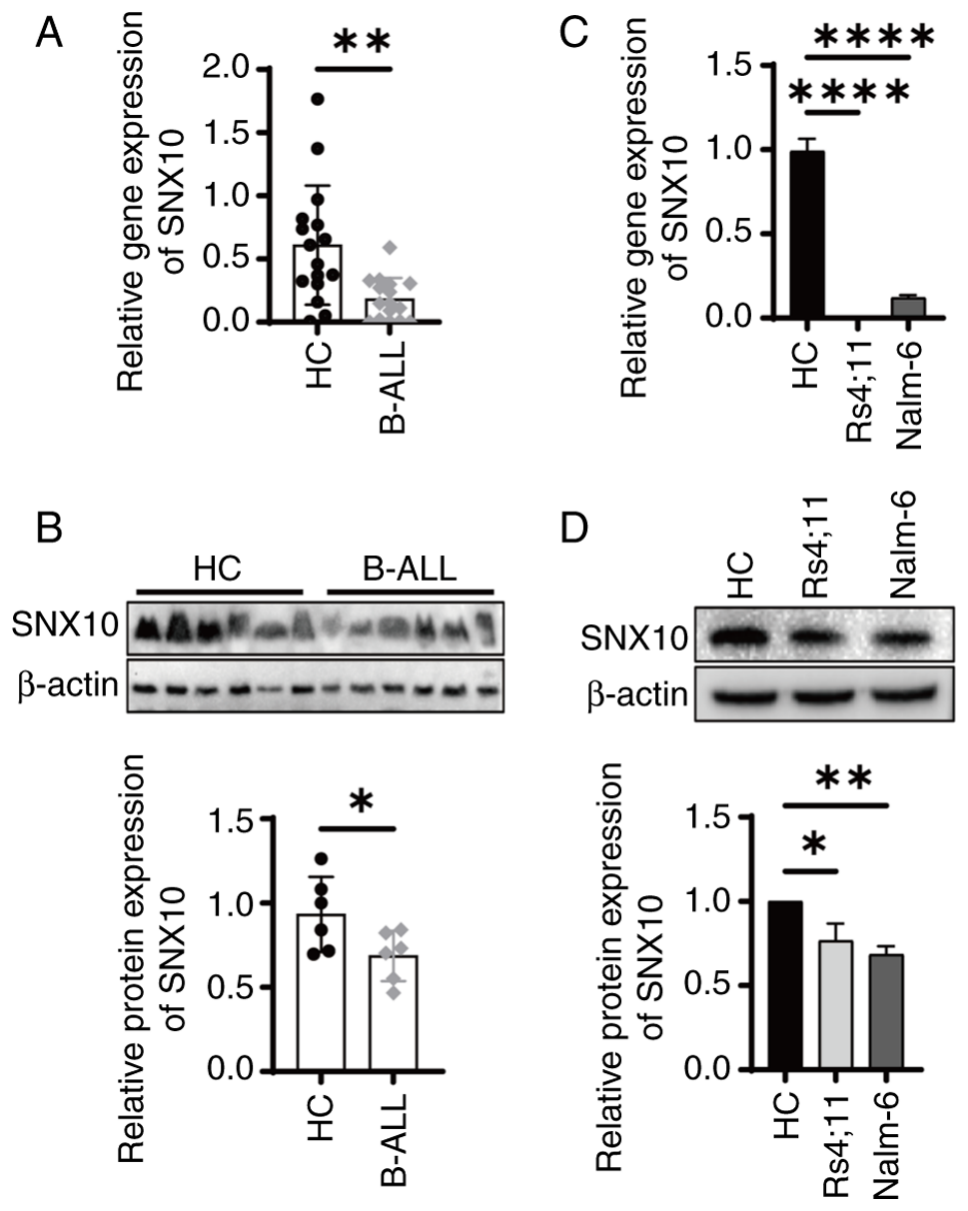
SNX10 expression levels decrease in patients with B-ALL and cell lines. (A) SNX10 (A) mRNA levels in BMMCs from healthy donors (n=18) and patients with primary B-ALL (n=18). (B) SNX10 protein levels in BMMCs from healthy donors (n=6) and patients with primary B-ALL (n=6). SNX10 (C) mRNA and (D) protein expression levels in RS4;11 and Nalm-6 cells (n=3). Data were compared using an unpaired Student's t-test or a one-way ANOVA. Data are presented as the mean ± SD. *P<0.05, **P<0.01 and ****P<0.0001 vs. the HC group. SNX10, sorting nexin 10; B-ALL, B-cell acute lymphoblastic leukemia; HC, healthy control; BMMC, bone marrow mononuclear cell.

SNX10 influences cell proliferation and survival by regulating lysosomal function and vesicle transport, as well as processes such as apoptosis and autophagy (9), which are particularly critical for leukemia cells. However, its role in B-ALL remains to be investigated. To examine the role of SNX10 in B-ALL, BMMCs were collected from both patients with B-ALL and healthy individuals. Analysis revealed a significant reduction in SNX10 mRNA (Fig. 2A, P<0.01) and protein expression (Fig. 2B, P<0.05) in the patients with B-ALL, consistent with the results of bioinformatics analysis (Fig. 1E). Similar results were obtained with B-cell precursor leukemia cell lines Nalm-6 and RS4;11 (Fig. 2C and D).

### Knockdown of SNX10 inhibits the proliferation of B-ALL cells

**Figure 3.**
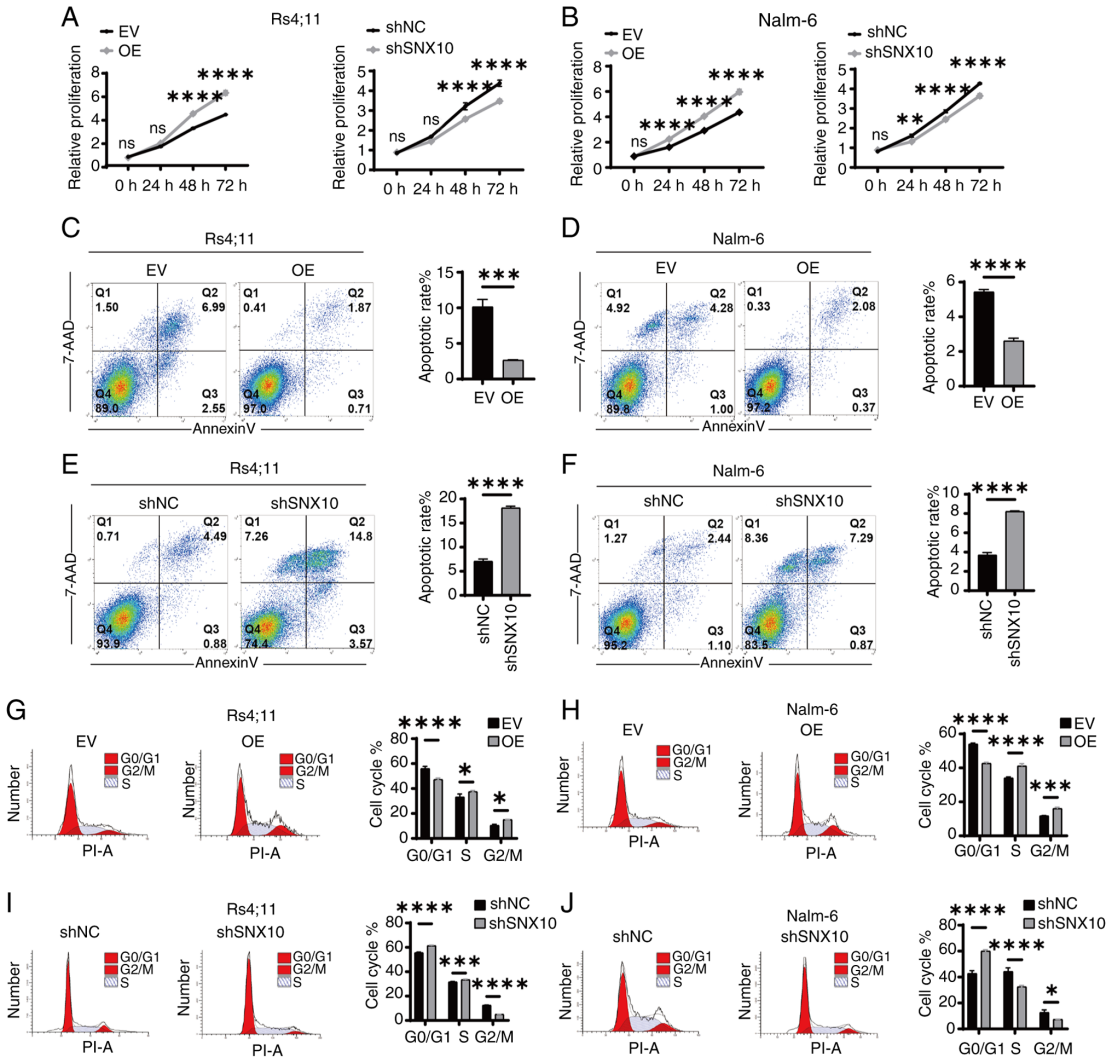
SNX10 regulates the proliferation of B-ALL cells through the regulation of apoptosis and cell cycle progression. Proliferation of SNX10-OE and knockdown (A) RS4;11 and (B) Nalm-6 cells was detected using Cell Counting Kit-8 assays. Apoptosis (annexin V/7-AAD staining) of SNX10-OE and knockdown (C and E) RS4;11 and (D and F) Nalm-6 cells was detected using flow cytometry. Cell cycle distribution analysis of SNX10-OE and knockdown (G and I) RS4;11 and (H and J) Nalm-6 cells. Data are presented as the mean ± SD of three independent repeats and compared using a one-way or two-way ANOVA. *P<0.05, **P<0.01, ***P<0.001 and ****P<0.0001 vs. EV group or shNC group. SNX10, sorting nexin 10; B-ALL, B-cell acute lymphoblastic leukemia; OE, overexpression; EV, empty vector; shRNA, short hairpin RNA; NC, negative control; ns, not significant.

To further investigate the function of SNX10 in B-ALL cells, RS4;11 and Nalm-6 SNX10 overexpression and knockdown cells were established by lentiviral infection and confirmed through RT-qPCR (Fig. S1A, C, E and G) and western blotting (Fig. S1B, D, F and H). Among all the shRNA knockdown cell lines, shSNX10-1 exhibited the highest knockdown efficiency in both cell types and was thus used for further experiments. Then, increased cell proliferation was observed in both cell types with SNX10 overexpression, whereas knockdown of SNX10 expression inhibited cell proliferation (Fig. 3A and B). A decreased ratio of apoptosis (Fig. 3C and D) and an increased proportion of cells in the G2/M phase of the cell cycle (Fig. 3G and H) were observed in these SNX10-overexpressing cells. By contrast, SNX10-knockdown cells exhibited increased apoptosis (Fig. 3E and F) and a higher proportion of cells arrested in the G0/G1 phase (Fig. 3I and J). These findings suggest that SNX10 plays a role in the proliferation of B-ALL cells by affecting their apoptosis and cell cycle progression.

### SNX10 promotes the development of B-ALL cells via regulation of the PI3K/Akt pathway

**Figure 4.**
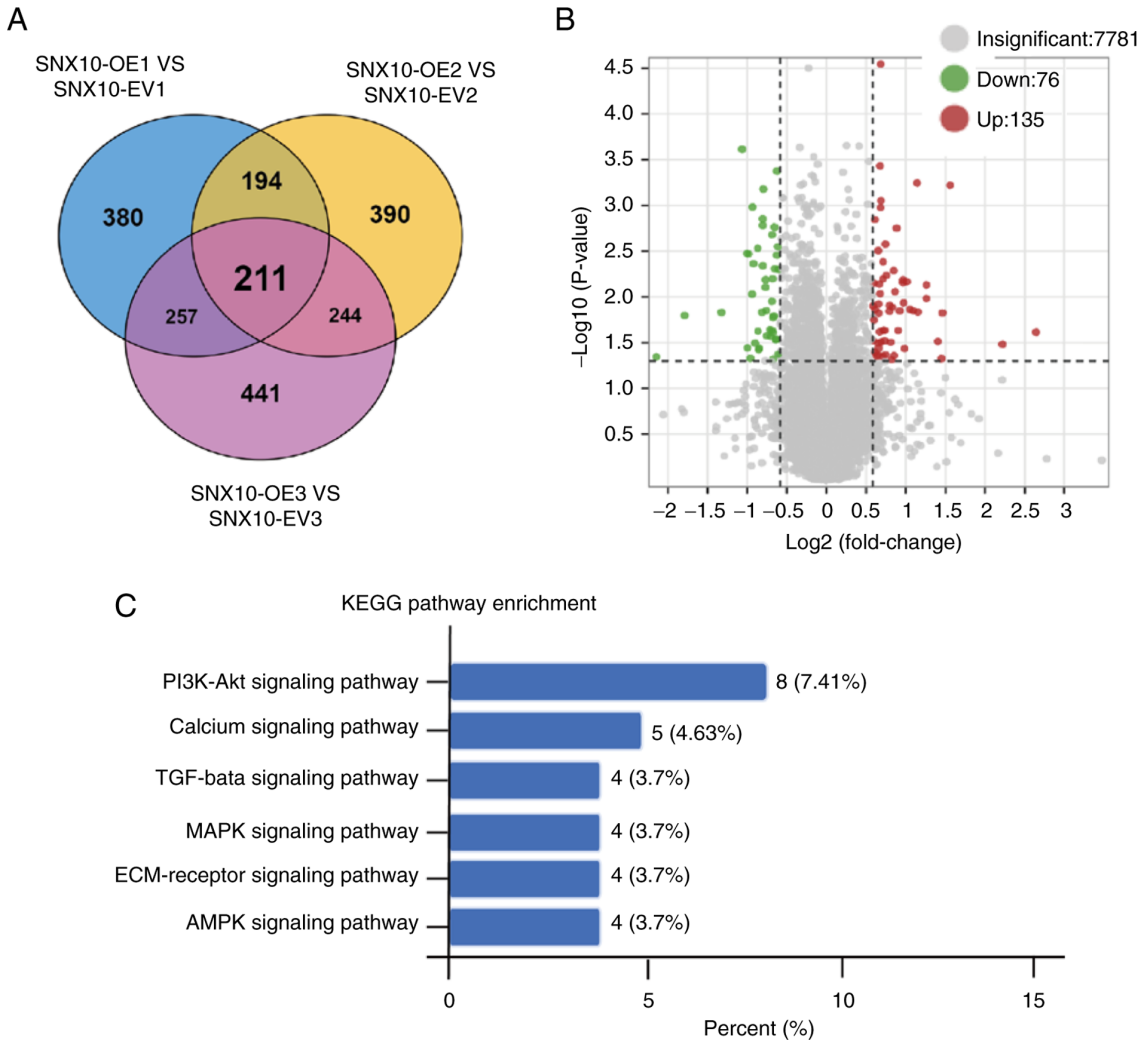
SNX10 promotes B-ALL cellular behaviors via the PI3K/Akt signaling pathway. (A) Venn diagram showing the 211 significantly differentially expressed proteins identified between SNX10-OE Rs4;11 and EV cells. (B) Volcano plot of the differentially expressed proteins showed 76 downregulated and 135 upregulated proteins. (C) KEGG pathway enrichment analysis of the differentially expressed proteins. SNX10, sorting nexin 10; B-ALL, B-cell acute lymphoblastic leukemia; OE, overexpression; EV, empty vector; KEGG, Kyoto Encyclopedia of Genes and Genomes.

To investigate the molecular mechanisms underlying the role of SNX10 in B-ALL, proteomic sequencing on cytoplasmic and membrane proteins extracted from SNX10-overexpressing Rs4;11 cells and their corresponding EV cells were performed. A total of 211 differentially expressed proteins were identified (Fig. 4A), of which 76 were downregulated and 135 were upregulated (Fig. 4B). To further elucidate the pathways influenced by these differentially expressed proteins, KEGG pathway enrichment analysis was performed. The PI3K-Akt signaling pathway, calcium signaling pathway, TGF-β signaling pathway, MAPK signaling pathway, ECM-receptor signaling pathway and AMPK signaling were all enriched (Fig. 4C). Among these, the PI3K-Akt pathway showed the most notable changes, suggesting that SNX10 may influence the properties of B-ALL cells via the PI3K-Akt signaling pathway.

### SNX10 regulates the proliferation, apoptosis and cycle progression of B-ALL cells via the PI3K/AKT signaling pathway

**Figure 5.**
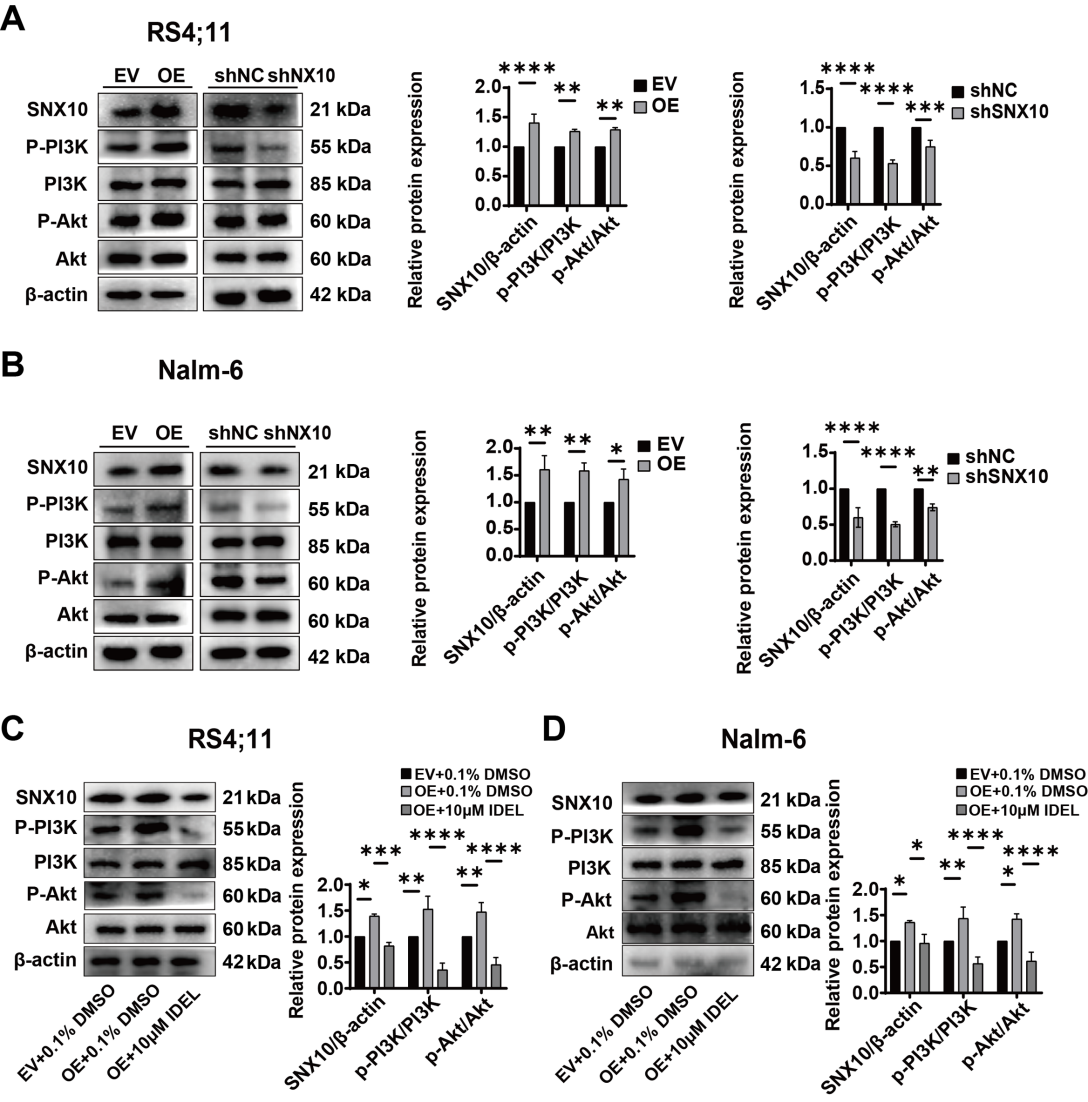
SNX10 influences the activity of the PI3K/Akt signaling pathway in B-ALL cells. The protein expression levels of PI3K, p-PI3K, Akt and p-Akt proteins in SNX10-OE and knockdown (A) Rs4;11 and (B) Nalm-6 cells were detected. After treating SNX10-OE B-ALL cells with 10 µM IDEL (a PI3K inhibitor) for 24 h, the protein expression levels of PI3K, p-PI3K, Akt and p-Akt proteins in SNX10-OE and knockdown (C) Rs4;11 and (D) Nalm-6 cells were detected. Data are presented as the mean ± SD of three independent repeats and compared using a one-way or two-way ANOVA. *P<0.05, **P<0.01, ***P<0.001 and ****P<0.0001 vs. EV group or shNC group. SNX10, sorting nexin 10; B-ALL, B-cell acute lymphoblastic leukemia; OE, overexpression; EV, empty vector; shRNA, short hairpin RNA; NC, negative control; IDEL, Idelalisib; p-, phosphorylated.

**Figure 6.**
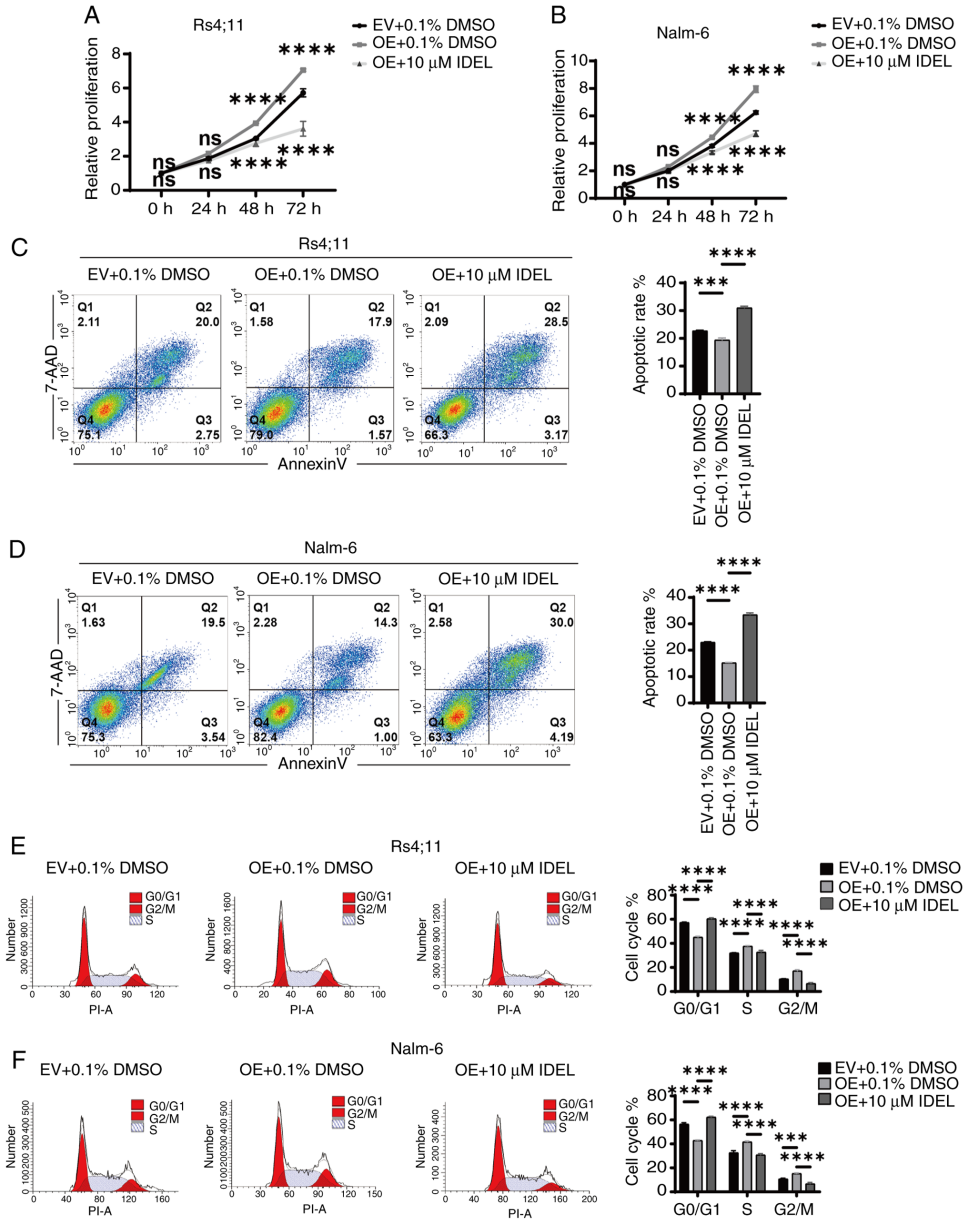
SNX10 affects the activity of B-ALL cells through the PI3K/Akt signaling pathway. After treating (A) Rs4;11 cells and (B) Nalm-6 cells with 10 µM IDEL (a PI3K inhibitor) for 24 h, cell proliferation was analyzed using a Cell Counting Kit-8 assay. Apoptosis of (C) Rs4;11 cells and (D) Nalm-6 cells (annexin V/7-AAD staining) was analyzed by flow cytometry. Cell cycle distribution in (E) Rs4;11 cells and (F) Nalm-6 cells was analyzed by flow cytometry. Data are presented as the mean ± SD of three independent repeats and compared using a one-way or two-way ANOVA. ***P<0.001 and ****P<0.0001 vs. EV group. SNX10, sorting nexin 10; B-ALL, B-cell acute lymphoblastic leukemia; OE, overexpression; EV, empty vector; IDEL, Idelalisib; ns, not significant.

The expression of key molecules within the PI3K-Akt signaling pathway in SNX10-overexpressing and knockdown Nalm-6 and Rs4;11 cells was next assessed. The overexpression of SNX10 resulted in increased phosphorylation of PI3K and Akt, and significantly elevated ratios of p-PI3K/PI3K and p-Akt/Akt in both cell lines (Fig. 5A and B), indicating the activation of the PI3K-Akt signaling pathway. Conversely, SNX10 knockdown exhibited reduced phosphorylation of PI3K and Akt proteins, resulting in a decrease in the ratios of p-PI3K/PI3K and p-Akt/Akt (Fig. 5A and B). To further confirm the role of SNX10 in activating the PI3K-Akt pathway, SNX10-overexpressing cells were treated with 10 µM IDEL, a PI3K inhibitor (32), and observed decreased phosphorylation of PI3K and Akt, and concurrently a reduction in the ratio of p-PI3K/PI3K and p-Akt/Akt (Fig. 5C and D). This inhibition also counteracted the effects of SNX10 overexpression, namely, reduced cell proliferation (Fig. 6A and B), increased apoptosis (Fig. 6C and D), and a decreased proportion of cells in the G2/M phase (Fig. 6E and F). These findings indicate that SNX10 regulates apoptosis and cell cycle progression by modulating the PI3K-Akt signaling pathway, promoting the proliferation of B-ALL.

### SNX10 overexpression accelerates the development of B-ALL in a xenograft mouse model

**Figure 7.**
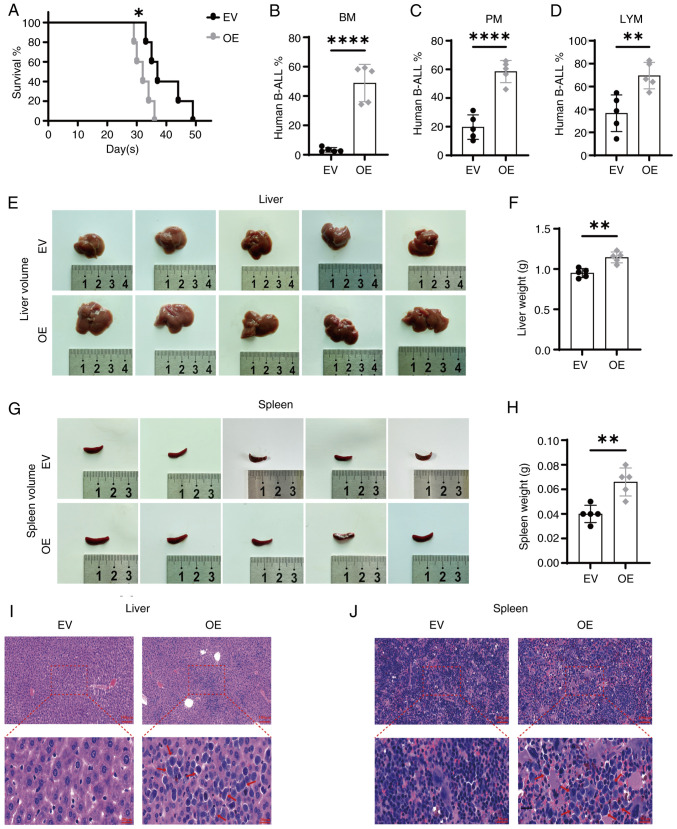
Overexpression of SNX10 accelerates the progression of B-ALL in a CDX xenograft mouse model. (A) Survival was analyzed using the Kaplan-Meier method. The presence of human B-ALL cells (human CD19-APC/GFP-FITC) in (B) BM, (C) PM and (D) LYM cells were assessed using flow cytometry. The liver (E) volume, (F) weight, and spleen (G) volume and (H) weight were measured. Tissue sections were stained with hematoxylin-eosin, and the leukemia infiltration areas are indicated with the red arrow, in the (I) liver and (J) spleen. Scale bar, 100 µm (top) and 20 µm (bottom). Data are presented as the mean ± SD of three independent repeats and compared using a Student's t-test. *P<0.05, **P<0.01 and ****P<0.0001 vs. EV group. SNX10, sorting nexin 10; B-ALL, B-cell acute lymphoblastic leukemia; OE, overexpression; EV, empty vector; BM, bone marrow; PM, peripheral blood; LYM, lymph node.

To further validate the *in vitro* findings that high levels of SNX10 promoted the growth and proliferation of leukemia cells, a CDX xenograft model of B-ALL was established using NOD-SCID mice via tail-vein injection of SNX10-overexpressing RS4;11 cells or EV cells. By day 21, mice injected with the SNX10-overexpressing cells began to display symptoms related to B-ALL, including systemic tremors, hind limb weakness and gradual weight loss. The OS time of these mice was notably shorter compared with their controls (Fig. 7A, P<0.05), consistent with the results of the bioinformatics analysis (Fig. 1F). In addition, the proportion of human B-ALL cells was significantly higher in the bone marrow (Fig. 7B, P<0.0001), peripheral blood (Fig. 7C, P<0.0001) and lymph nodes (Fig. 7D, P<0.01) of mice injected with SNX10-overexpressing cells. Furthermore, the liver and spleen in these mice were also markedly enlarged in both volume (Fig. 7E and G) and weight (Fig. 7F and H, P<0.01). Additionally, an increased proportion of infiltrating leukemia cells was observed in the liver (Fig. 7I) and spleen (Fig. 7J) of these mice. These results collectively suggest that elevated SNX10 levels facilitate the proliferation, migration and infiltration of B-ALL cells, thereby accelerating leukemia progression *in vivo*.

## Discussion

As a relatively recently identified cancer regulator (13,14,35), the specific mechanism of action of SNX10 in B-ALL has not been assessed previously. The present study addressed this gap by investigating the role of SNX10 in B-ALL and its potential mechanisms. It was found that upregulated expression of SNX10 activated the PI3K-Akt signaling pathway, thereby reducing apoptosis and increasing the proportion of cells in the G2/M phase, thus promoting the proliferation of B-ALL cells and accelerating disease progression. By contrast, SNX10 was generally downregulated in the samples of patients with B-ALL, as well as in B-ALL cell lines. Knockdown of SNX10 resulted in reduced PI3K-Akt signaling, an increased proportion of cells in the G0/G1 phase and increased apoptosis, thus reducing the proliferation of B-ALL cells. The *in vivo* experiments further supported these findings, illustrating that high SNX10 expression contributed to aggressive behaviors of B-ALL.

High expression of SNX10 and poorer prognosis in B-ALL is a trend also observed in cervical cancer (14) and glioblastoma (15). The expression levels of SNX10 differentially affect the progression and prognosis of various tumors. For example, in osteosarcoma tissues (36), high expression of SNX10 inhibits the proliferation, migration and invasion of tumor cells. Additionally, low expression of SNX10 has been associated with a poor prognosis in patients with stomach adenocarcinoma (37) and colorectal cancer (13). These observations suggest that SNX10 may act as a bifunctional gene, with its effects depending on tissue type and cellular context. The impact of SNX10 on B-ALL also appears to depend on its expression level within the context of the specific disease; however, the specific molecular mechanisms require further experimental exploration.

SNX10 has been demonstrated to be involved in several cellular processes, including intracellular transport (38) and signal transduction (15), as well as in physiological processes such as cellular absorption and secretion (10,11), and intracellular glucose metabolism (12). In healthy populations, SNX10 is expressed at high levels, while its expression is reduced in patients with B-ALL, which may reflect specific metabolic or transcriptional regulatory changes in leukemia cells. This decrease in expression may be related to mechanisms that inhibit the proliferation, survival, or drug resistance of leukemia cells *in vivo*. Numerous studies have shown that not only tumor cells (39) but also immune cells (40) in the tumor microenvironment undergo metabolic reprogramming, which may directly affect the biological activity of tumor cells and is closely related to tumor drug resistance and malignant progression (41). In B-ALL, the expression of SNX10 may be influenced by the tumor microenvironment, leading to its reduced expression in tumor cells. Although certain patients with B-ALL exhibit relatively high levels of SNX10 expression, this high expression does not necessarily indicate a functionally ‘healthy’ state. Conversely, aberrantly relatively upregulated SNX10 expression may be associated with cell proliferation, an anti-apoptotic effect, or treatment resistance, resulting in a poor prognosis. In the present study, both *in vivo* and *in vitro* experiments demonstrated that overexpression of SNX10 modified the cell cycle of leukemia cells, suppressed apoptosis, and promoted cell survival. Upregulated expression of SNX10 may signify a highly active state of tumor cells, associated with increased aggressiveness or a higher probability of recurrence. Therefore, despite the elevated expression levels of SNX10 observed in healthy individuals, its expression pattern and impact in leukemia may be associated with tumor progression, exhibiting a duality in its effects.

The present study is the first to demonstrate that SNX10 regulates the proliferation, apoptosis and cell cycle processes of B-ALL cells via the PI3K/Akt signaling pathway, to the best of our knowledge. This finding suggests that SNX10 may play an important role in the development of B-ALL and could be associated with the onset of leukemia, resistance to treatment and a poorer prognosis. Consequently, SNX10 has the potential to serve as a biomarker for B-ALL and a therapeutic target for drug development. For example, by evaluating the expression levels of SNX10 across a range of populations of patients with B-ALL, high-risk groups can be identified, and subsequent personalized treatment plans can be formulated (42,43). Alternatively, by developing oral nanoparticles of SNX10-shRNA plasmids (44,45) or through the use of an SNX10 protein-protein interaction inhibitor such as DC-SX029 (46) in combination with existing treatment regimens, particularly targeted drugs such as PI3K/Akt inhibitors (5,14,32), it may be possible to overcome resistance and enhance therapeutic efficacy. Therefore, further research into the specific mechanisms of SNX10 in B-ALL cells is warranted. By exploring the interactions between SNX10 and the PI3K/Akt signaling pathway in-depth, novel therapeutic targets may be uncovered. Additionally, it is also worth investigating whether the function of SNX10 is regulated by other cellular signaling pathways, which can aid in constructing a more comprehensive model of the pathogenesis of B-ALL and promote the implementation of personalized treatment.

B-ALL is a genetically heterogeneous disease (47). The limited variety of cell lines and clinical samples utilized in the present study may hinder the applicability and statistical relevance of the results. Future research should aim to enhance the sample size, especially in multicenter clinical trials, to confirm the role of SNX10 across patients from different demographic backgrounds. Furthermore, the effects of targeting SNX10 in normal cells were not evaluated, necessitating further studies to assess the selectivity of its therapeutic targets to reduce potential side effects. Additionally, the present study used a NOD-SCID mouse model (48) to investigate the role of SNX10 *in vivo*. While this model is widely used in cancer research due to its lack of functional T and B cells (49), allowing human cell transplantation, it may not fully recapitulate the human tumor microenvironment, especially in terms of immune interactions. B-ALL progression and treatment responses are complex processes influenced by the immune system, and the absence of immune cells in NOD-SCID mice limits the study of these interactions. Furthermore, the mouse microenvironment differs from the human setting, potentially affecting B-ALL cell behavior and limiting clinical relevance. Although the NOD-SCID model is valuable for drug screening and mechanistic research, future studies may benefit from employing more humanized models, such as humanized mice (50) or transgenic models (51), to simulate the immune interactions and microenvironmental conditions more accurately. Cross-validation with multiple models may enhance the reliability of these findings and facilitate their clinical translation.

In conclusion, the present study identified SNX10 as a core hub gene within the B-ALL-related signaling network and revealed its significant impact on B-ALL cell biology. Through both *in vitro* and *in vivo* experiments, it was found that SNX10 regulated the PI3K/Akt signaling pathway, affecting B-ALL cell proliferation, apoptosis, cell cycle progression, and disease progression. These findings provide novel insights into the potential of SNX10 as a prognostic marker and therapeutic target for the management of B-ALL, supporting further research into SNX10-modulating therapies that could improve treatment outcomes for patients with B-ALL.

## Supplementary Material

Supporting Data

Supporting Data

Supporting Data

Supporting Data

Supporting Data

## Data Availability

The data generated in the present study may be found in the ProteomeXchange Consortium under accession number PXD061830 or at the following URL: http://proteomecentral.proteomexchange.org/cgi/GetDataset?ID=PXD061830. The data generated in the present study may be requested from the corresponding author.
