# Supplementary material for: SNX10 regulates the proliferation, apoptosis and cell cycle of acute B lymphoblastic leukemia cells via the PI3K/Akt signaling pathway
Source: Oncol Rep. 2025 May 8;54(1):78. doi: 10.3892/or.2025.8911 (PMC12093086; doi:10.3892/or.2025.8911)

Figure S1. Establishment of stable SNX10-OE and knockdown RS4;11 and Nalm-6 cells. (A) mRNA and (B) protein expression levels of SNX10 in the SNX10-OE RS4;11 cells. (C) mRNA and (D) protein expression levels of SNX10 in the SNX10 knockdown RS4;11 cells. (E) mRNA and (F) protein expression levels of SNX10 in the SNX10-OE Nalm-6 cells. (G) mRNA and (H) protein expression levels of SNX10 in the SNX10 knockdown Nalm-6 cells. Data are presented as the mean ± SD of three independent repeats and compared using a Student's t-test. *P<0.05, **P<0.01, ***P<0.001 and ****P<0.0001 vs. EV group or shNC group. SNX10, sorting nexin 10; OE, overexpression; EV, empty vector; shRNA, short hairpin RNA; NC, negative control; ns, not significant.

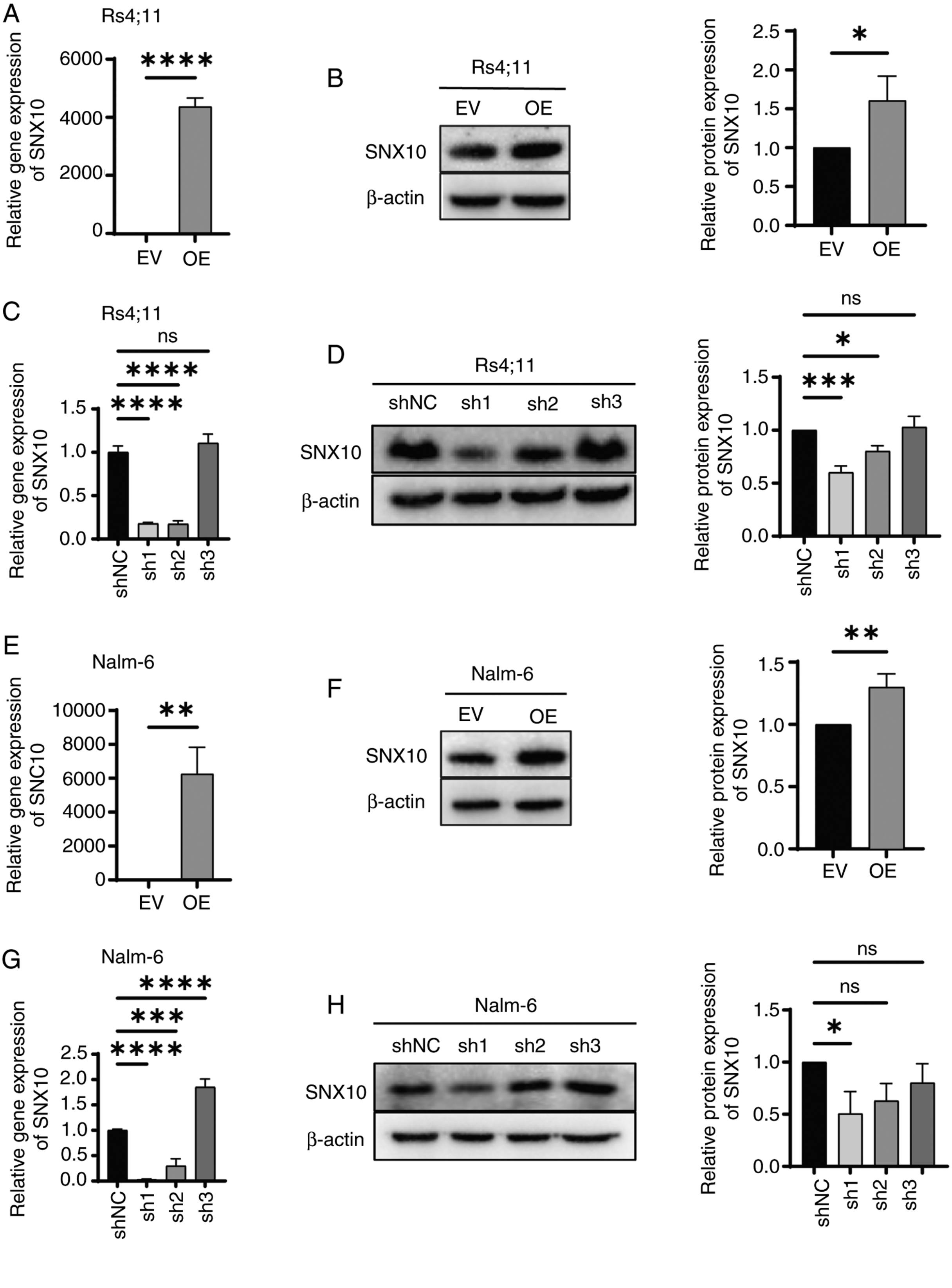

Supplement: Supporting Data [file Supplementary_Data1.pdf]
