# Supplementary material for: SNX10 regulates the proliferation, apoptosis and cell cycle of acute B lymphoblastic leukemia cells via the PI3K/Akt signaling pathway
Source: Oncol Rep. 2025 May 8;54(1):78. doi: 10.3892/or.2025.8911 (PMC12093086; doi:10.3892/or.2025.8911)
Supplement: Supporting Data [file Supplementary_Data2.pdf]

Table SI. The patients with B-ALL samples characteristic.

Characteristics	Number	Percentage (%)
Sex		
Male	10	41.7
Female	14	58.3
Age, years		
<18	14	58.3
18-34	6	25
35-80	4	16.4
White blood cells count (cells x10^9 /l)		
<30	14	58.3
30-99	7	29.2
≥100	3	12.5
Immunophenotype		
Common-B-ALL	19	79.2
Pre-B-ALL	5	20.8
Fusion genes		
BCR-ABLp190 (positive)	6	25
E2A-PBX1	2	8.3
ETV6：RUNX1	1	4.2
TCF:PBX1	2	8.3
P2RY8/CRLF2	2	8.3
CDKN2A-MTAP	1	4.2

B-ALL, B-cell acute lymphoblastic leukemia.

Table SII. Plasmid sequence.

Sequence name	Gene sequence
Homo-SNX10 (Overexpression)	GAATTCGCCACCATGTTTCCGGAACAACAGAAAGAGGAATTTGTAAGTGTCTGGGTTCGAGATCCTAGGATTCAGAAGGAGGACTTCTGGCATTCTTACATTGACTATGAGATATGTATTCATACTAATAGCATGTGTTTTACAATGAAAACATCCTGTGTACGAAGAAGATATAGAGAATTCGTGTGGCTGAGGCAGAGACTCCAAAGTAATGCGTTGCTGGTACAACTGCCAGAACTTCCATCTAAAAACCTGTTTTTCAACATGAACAATCGCCAGCACGTGGATCAGCGTCGCCAGGGTCTGGAAGATTTCCTCAGAAAAGTCCTACAGAATGCACTTTTGCTTTCAGATAGCAGCCTTCACCTCTTCTTACAGAGCCATCTGAATTCAGAAGACATTGAGGCGTGTGTTTCTGGGCAGACTAAGTACTCTGTGGAAGAAGCAATTCACAAGTTTGCCTTAATGAATAGACGTTTCCCTGAAGAAGATGAAGAAGGAAAAAAAGAAAATGATATAGATTATGATTCAGAAAGTTCATCCTCTGGGCTTGGACACAGTAGTGATGACAGCAGTTCACATGGATGTAAAGTAAATACAGCTCCGCAGGAATCCTGAGGATCC
empty vector	pCDH-CMV-MCS-EF1a-copGFP-T2A-Puro
sh#1	5’-CCGGGCAATTCACAAGTTTGCCTTACTCGAGTAAGGCAAACTTGTGAATTGCTTTTTGAATT-3
sh#2	5’-CCGGGCAGAGACTCCAAAGTAATGCCTCGAGGCATTACTTTGGAGTCTCTGCTTTTTGAATT-3’
sh#3	5’-CCGGGCAGTTCACATGGATGTAAAGCTCGAGCTTTACATCCATGTGAACTGCTTTTTGAATT-3’
sh-negative control	pLKO.1-U6-scramble-EF1a-copGFP-T2A-puro<br>5’-CCTAAGGTTAAGTCGCCCTCGCTCGAGCGAGGGCGACTTAACCTTAGG-3’

sh-, short hairpin.
